# Analysis of the Trends in Publications on Clinical Cancer Research in Mainland China from the Surveillance, Epidemiology, and End Results (SEER) Database: Bibliometric Study

**DOI:** 10.2196/21931

**Published:** 2020-11-17

**Authors:** Min-Qiang Lin, Chen-Lu Lian, Ping Zhou, Jian Lei, Jun Wang, Li Hua, Juan Zhou, San-Gang Wu

**Affiliations:** 1 Department of Scientific Management, The First Affiliated Hospital of Xiamen University Xiamen China; 2 Department of Radiation Oncology, The First Affiliated Hospital of Xiamen University Xiamen China; 3 Department of Obstetrics and Gynecology, The First Affiliated Hospital of Xiamen University Xiamen China

**Keywords:** cancer, China, data collection, bibliometrics, PubMed, SEER program

## Abstract

**Background:**

The application of China’s big data sector in cancer research is just the beginning. In recent decades, more and more Chinese scholars have used the Surveillance, Epidemiology, and End Results (SEER) database for clinical cancer research. A comprehensive bibliometric study is required to analyze the tendency of Chinese scholars to utilize the SEER database for clinical cancer research and provide a reference for the future of big data analytics.

**Objective:**

Our study aimed to assess the trend of publications on clinical cancer research in mainland China from the SEER database.

**Methods:**

We performed a PubMed search to identify papers published with data from the SEER database in mainland China until August 31, 2020.

**Results:**

A total of 1566 papers utilizing the SEER database that were authored by investigators in mainland China were identified. Over the past years, significant growth in studies based on the SEER database was observed (*P*<.001). The top 5 research topics were breast cancer (213/1566, 13.6%), followed by colorectal cancer (185/1566, 11.8%), lung cancer (179/1566, 11.4%), gastrointestinal cancer (excluding colorectal cancer; 149/1566, 9.5%), and genital system cancer (93/1566, 5.9%). Approximately 75.2% (1178/1566) of papers were published from the eastern coastal region of China, and Fudan University Shanghai Cancer Center (Shanghai, China) was the most active organization. Overall, 267 journals were analyzed in this study, of which Oncotarget was the most contributing journal (136/267, 50.9%). Of the 1566 papers studied, 585 (37.4%) were published in the second quartile, 489 (31.2%) in the third quartile, 312 (19.9%) in the first quartile, and 80 (5.1%) in the fourth quartile, with 100 (6.4%) having an unknown Journal Citation Reports ranking.

**Conclusions:**

Clinical cancer research based on the SEER database in mainland China underwent constant and rapid growth during recent years. High-quality and comprehensive cancer databases based on Chinese demographic data are urgently needed.

## Introduction

### Background

The incidence of human cancer is increasing worldwide. It is estimated that the global burden of cancer will increase by more than 60% by 2040 [1]. Cancer has been an important public health problem in low- and middle-income countries, as well as in upper-middle-income countries. Cancer research has become one of the leading research fields of bioscience around the world, and the number of publications on cancer increases at a rate of more than 2% per year [2].

A high-quality cancer database can provide researchers with convenient data analysis and build a sharing platform among researchers, which could pave the way for revealing the mechanism of tumorigenesis and its progression [3]. To share clinical data with different regions, some other countries plunged into building multicenter databases far ahead of China [4]. In 1973, the US National Cancer Institute combined the tumor registration stations in several regions to form the Surveillance, Epidemiology, and End Results (SEER) database. The SEER program is a globally accessible authoritative cancer database representing approximately 34.6% of the US population, which includes non-Hispanic White, non-Hispanic Black, Hispanic, and Asian populations [5]. The SEER program collects data on patient demographics, tumor location, tumor stage, first course of therapy, and vital status. The SEER database is a valuable, population-based resource that can be used to study the diagnosis and treatment across demographic characteristics and geographic areas, and it has become a unique research resource for oncology practice. It provides morbidity and mortality data on various histopathological subtypes, and data on molecular characteristics are also expanding. The database is being further developed to capture other biomarker data and the results of specific populations, and to expand the biobank to support cutting-edge cancer research that can improve oncology practices. Therefore, the SEER program plays an important role in clinical cancer research, public health management, and policy making [5].

In recent years, China has made significant progress in clinical cancer research, and many studies have gained international recognition [6-10]. Although major hospitals have established databases in China, they have not shared their research findings with one another. Since the SEER database is a globally accessible authoritative cancer database, more and more scholars, particularly those from China, have used it to conduct clinical cancer research in recent years. There are a lot of differences between the United States and China, including their population’s genetic makeup, health system, health services, health insurance, health policies, socioeconomic status, and culture. Therefore, the research findings in the SEER cancer registries may not be generalizable to the people of China. However, several recent studies found that the characteristics of the data from the SEER program were consistent with those from Chinese institutions [11-14]. There is currently no comprehensive bibliometric study that has characterized the clinical cancer research in China based on the SEER database. Carrying out a comprehensive bibliometric analysis is helpful to analyze the contribution of Chinese scholars to clinical cancer research and provide a specific clinical reference for the future of big data analytics.

### Objective

This study aimed to evaluate the characteristics of clinical cancer research using SEER data from mainland China using a bibliometric approach.

## Methods

### Search Strategy

Using the search terms “Surveillance, Epidemiology, and End Results or SEER, and China,” we identified related publications from the PubMed database before August 31, 2020. PubMed is a free, publicly available database established by the US National Library of Medicine [15]. As one of the largest databases in the life science and biomedical fields worldwide, it comprises more than 30 million biomedical abstracts from journals and online books.

Publications with first authors or corresponding authors affiliated with mainland Chinese institutions were included in the study, while those that utilized the SEER-Medicare database (SEER-Medicare data are not released outside of the United States) and special types of publications, including comments, letters, and reviews, were excluded. Papers from Taiwan Province, Hong Kong, and Macao Special Administrative Regions were also excluded from this analysis. This study was approved by the ethics committee of the First Affiliated Hospital of Xiamen University (Xiamen, Fujian, China).

### Indices

The indices analyzed in this study included the number and trend of publications, research topics, type of affiliation (university, hospital, or other research center), geographical distribution, journal, Journal Citation Reports (JCR) ranking, and status of international cooperation.

### Statistical Analysis

Descriptive statistical analyses were used in this study. Characteristics were evaluated and analyzed using Microsoft Excel, and linear regression was performed using SPSS statistical software (version 22.0; IBM Corp). *P* values <.05 were considered statistically significant.

## Results

### Number and Trend of Publications

**Figure 1:**
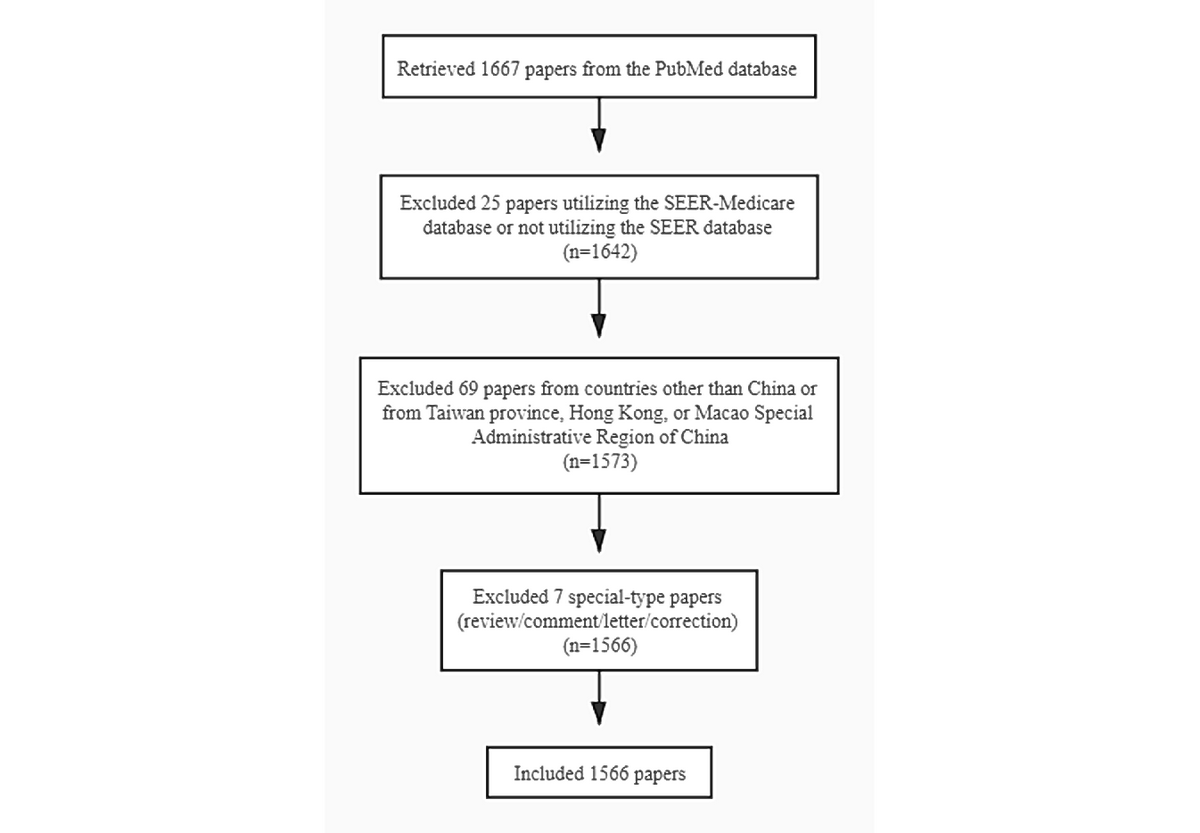
Flowchart of publication selection for the study.

**Figure 2:**
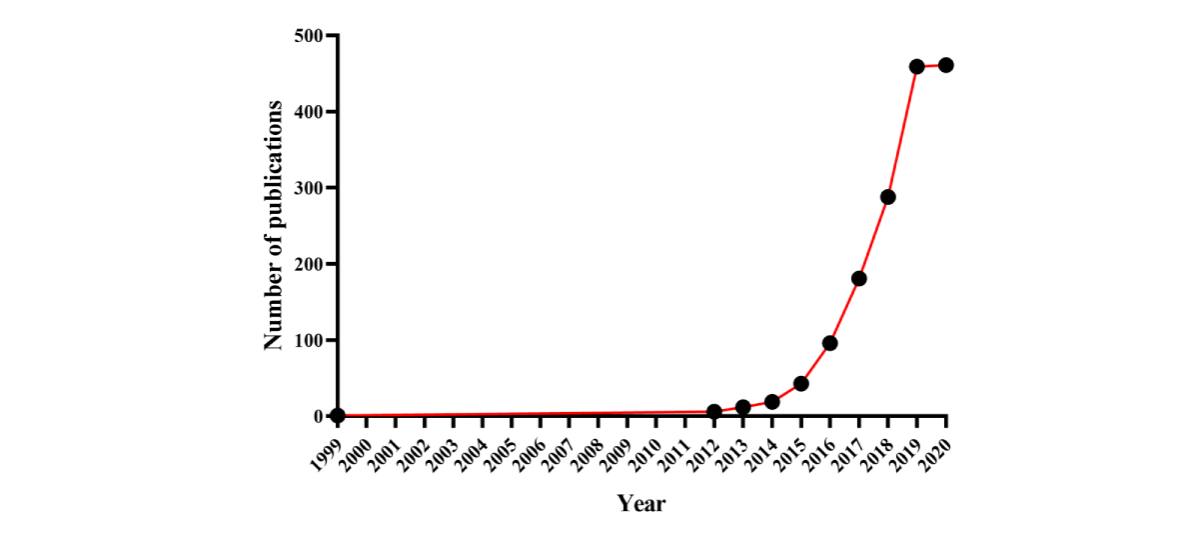
The trends in publications on clinical cancer research in mainland China from the Surveillance, Epidemiology, and End Results database between 1999 and 2020.

The flowchart of publication selection for this study is shown in Figure 1. We retrieved a total of 1667 publications, of which 1566 publications were included in this study. Figure 2 shows the trend in publications on clinical cancer research using the SEER database in mainland China (*R^2^*=0.430, *P*<.001). Chinese authors first used the SEER database in 1999. The single paper in the SEER database that was published in 1999 was authored by Guo et al [16], who were affiliated with the People’s Hospital of Beijing Medical University and had collaborated with Huvos and colleagues from Memorial Sloan-Kettering Cancer Center in the United States. Interestingly, there were no papers from Chinese institutions that utilized the SEER database during the 11 years from 2000 to the end of 2011. However, the number of papers published per year increased rapidly from 2012 to 2019: 2012 (n=6), 2013 (n=12), 2014 (n=19), 2015 (n=43), 2016 (n=96), 2017 (n=181), 2018 (n=288), and 2019 (n=459). Despite the COVID-19 pandemic in 2020, there were 461 publications as of August 31, 2020.

### Research Topics

Table 1 presents the main research topics of the 1566 papers included in the study. Breast cancer was the most frequently researched topic (213/1566, 13.6%), followed by colorectal cancer (185/1566, 11.8%), lung cancer (179/1566, 11.4%), gastrointestinal cancer (excluding colorectal cancer; 149/1566, 9.5%), and genital system cancer (93/1566, 5.9%). These top 5 research topics were the focus of 52.3% (819/1566) of the included papers.

**Figure 3:**
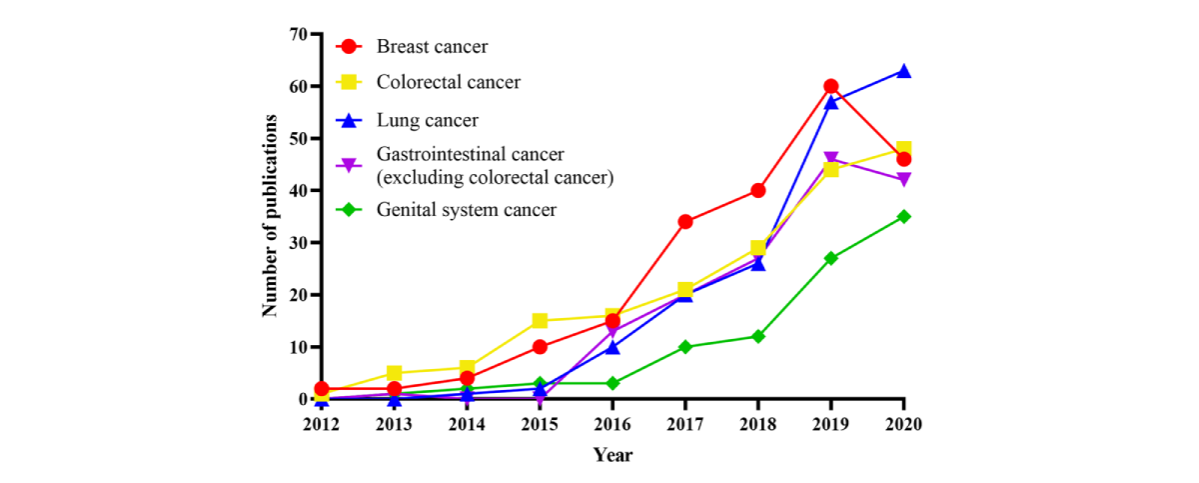
The trends in publications on the top 5 cancer sites in mainland China from the Surveillance, Epidemiology, and End Results database between 2012 and 2020.

Figure 3 shows the publication trends on the top 5 cancer sites in mainland China over time. It shows an increasing interest in research exploring breast cancer, lung cancer, gastrointestinal cancer (excluding colorectal cancer), colorectal cancer, and genital system cancer (in both genders). Chinese researchers were also studying rare cancers. A total of 164 papers discussed rare cancers, such as pulmonary lymphoepithelioma-like carcinoma, spindle cell carcinoma, thymoma, and adenoid cystic carcinoma of the breast, and this number was expanding rapidly in recent years.

**Table 1:** Main cancer research topics of publications in mainland China identified in the Surveillance, Epidemiology, and End Results database.

Main cancer research topics	Number of publications
Breast cancer	213
Colorectal cancer	185
Lung cancer	179
Gastrointestinal cancer (excluding colorectal cancer)	149
Genital system cancer	93
Pancreatic cancer	83
Liver cancer	72
Thyroid cancer	62
Esophageal cancer	62
Bone cancer	49

### Geographical Distribution of Publications

**Figure 4:**
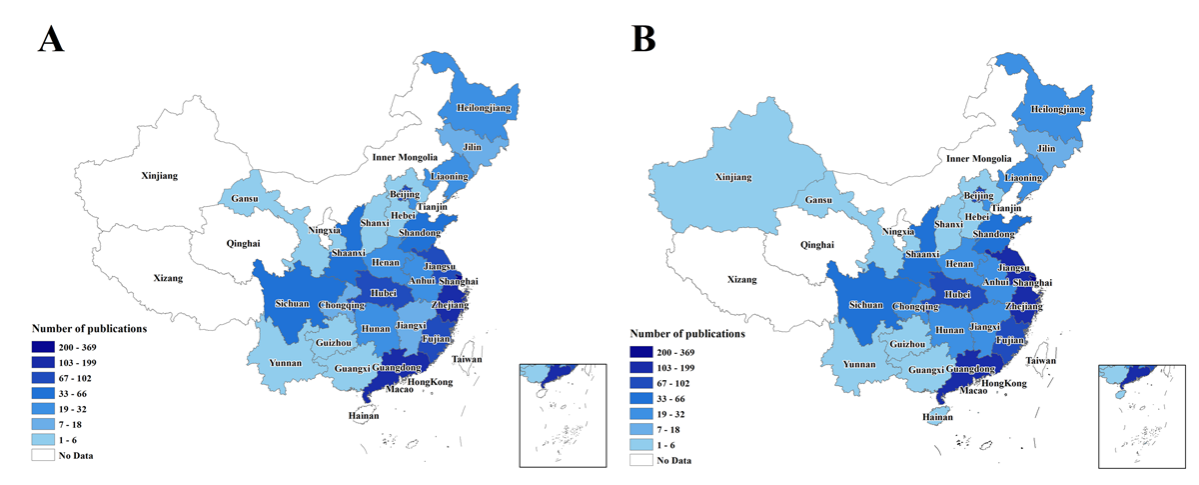
The distribution of the publications stratified by first authors (A) and corresponding authors (B) using the Surveillance, Epidemiology, and End Results database.

Figure 4A shows the distribution of the publications using the SEER database whose first authors were affiliated with Chinese institutions. These first authors who used the SEER database to publish papers came from 25 provinces and municipalities of China. Among all of these regions, first authors were most often affiliated with institutions in Shanghai (369/1566, 23.6%), followed by Guangdong (199/1566, 12.7%), Zhejiang (174/1566, 11.1%), Hubei (102/1566, 6.5%), and Jiangsu (102/1566, 6.5%).

Figure 4B shows the distribution of publications using the SEER database whose corresponding authors were affiliated with Chinese institutions. These corresponding authors who used the SEER database to publish papers came from 27 provinces or municipalities of China. A similar distribution was observed in the papers whose corresponding authors were affiliated with Chinese institutions, mostly concentrated in Shanghai, Guangdong, Zhejiang, Jiangsu, and Hubei.

### Top 10 Most Contributing Author Affiliations

The top 10 affiliated organizations of the most contributing first authors and corresponding authors extracted from the 1566 publications are presented in Tables 2 and 3. More than 75% (1178/1566) of papers were published from the eastern coastal region of China, Overall, the organization that was affiliated with the top contributing first authors was Fudan University Shanghai Cancer Center (163/1566, 10.4%), followed by Sun Yat-sen University Cancer Center (73/1566, 4.7%), the First Affiliated Hospital of Xi’an Jiaotong University (48/1566, 3.1%), West China Hospital of Sichuan University (42/1566, 2.7%), the Second Affiliated Hospital of Zhejiang University School of Medicine (41/1566, 2.6%), and the First Affiliated Hospital of Xiamen University (41/1566, 2.6%). All affiliations of first authors extracted from the 1566 publications (excluding three papers whose first authors were affiliated with the United States) were classified into three sectors (university, hospital, and government agency). The most contributing institution sector was hospital (1501/1563, 96.0%), followed by university (60/1563, 3.8%), and government agency (2/1563, 0.1%). Of the 1501 papers from hospitals, 1459 (97.2%) publications were produced from the best tertiary hospitals, and 1475 (98.3%) were from hospitals affiliated with universities.

The organization that was affiliated with the top contributing corresponding authors was Fudan University Shanghai Cancer Center (165/1566, 10.5%), followed by Sun Yat-sen University Cancer Center (77/1566, 4.9%), Zhongshan Hospital of Fudan University (43/1566, 2.7%), the First Affiliated Hospital of Xi’an Jiaotong University (43/1566, 2.7%), and the Second Affiliated Hospital of Zhejiang University School of Medicine (42/1566, 2.7%). All affiliations of corresponding authors extracted from the 1566 papers (excluding 35 publications’ organization affiliations of corresponding authors affiliated with the United States, Germany, Australia, and Japan) were also classified into three sectors (university, hospital, and government agency). Similarly, the most contributing institution sector was hospital (1474/1531, 96.3%), followed by university (54/1531, 3.5%), and government agency (3/1531, 0.2%). In the 1474 papers whose corresponding authors came from hospitals, 1440 (97.7%) publications were from best tertiary hospitals, and 1458 (98.9%) were from hospitals affiliated with universities.

**Table 2:** Top 10 affiliated organizations of the most contributing first authors.

Affiliations of first authors	Number of publications
Fudan University Shanghai Cancer Center	163
Sun Yat-sen University Cancer Center	73
The First Affiliated Hospital of Xi’an Jiaotong University	48
West China Hospital of Sichuan University	42
The Second Affiliated Hospital of Zhejiang University	41
The First Affiliated Hospital of Xiamen University	41
Zhongshan Hospital of Fudan University	39
Affiliated Union Hospital of Fujian Medical University	33
Union Hospital of Tongji Medical College of Huazhong University of Science and Technology	32
Renmin Hospital of Wuhan University	27

**Table 3:** Top 10 affiliated organizations of the most contributing corresponding authors.

Affiliations of corresponding authors	Number of publications
Fudan University Shanghai Cancer Center	165
Sun Yat-sen University Cancer Center	77
The First Affiliated Hospital of Xi’an Jiaotong University	43
Zhongshan Hospital of Fudan University	43
The Second Affiliated Hospital of Zhejiang University	42
West China Hospital of Sichuan University	42
Union Hospital of Tongji Medical College of Huazhong University of Science and Technology	33
Affiliated Union Hospital of Fujian Medical University	31
Renmin Hospital of Wuhan University	27
Shandong Cancer Hospital Affiliated to Shandong University	27

### Journals and Journal Visibility

**Figure 5:**
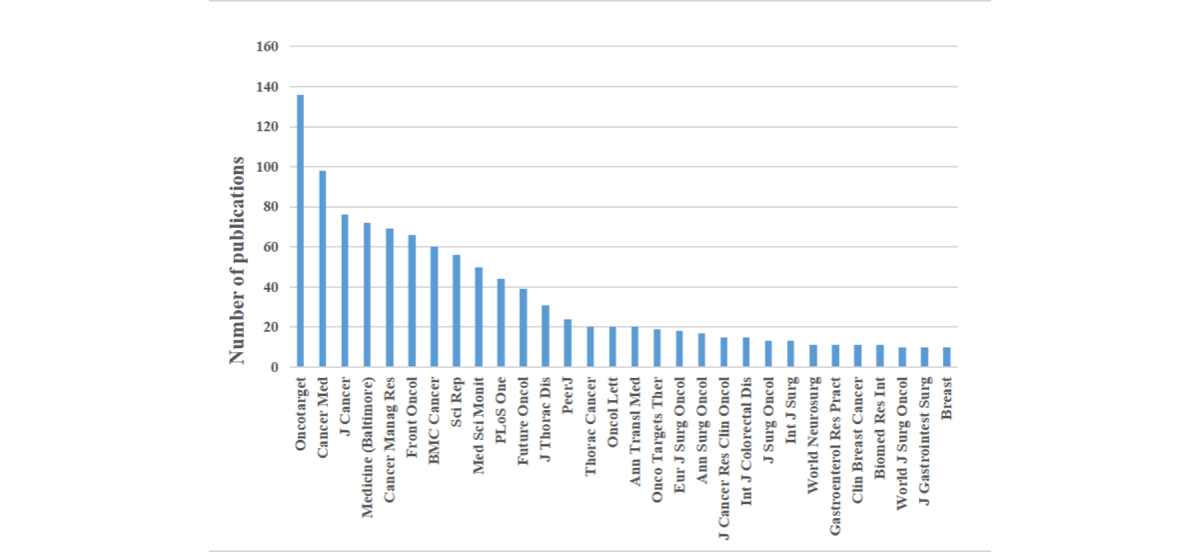
The distribution of the publications stratified by the top 30 most common journals.

**Figure 6:**
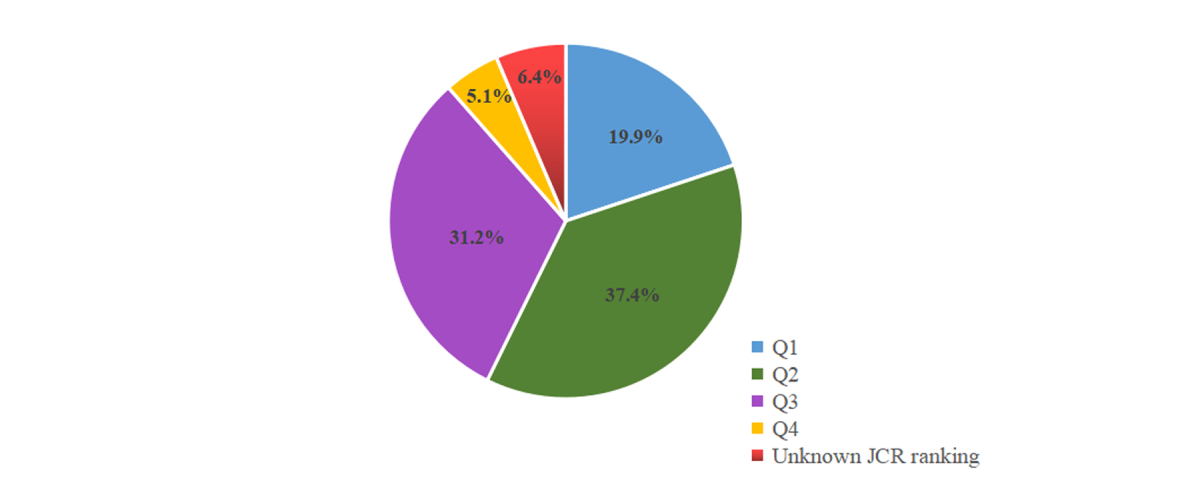
The distribution of the 1566 papers stratified by journal rankings published from Journal Citation Reports (JCR). Q1: first quartile; Q2: second quartile; Q3: third quartile; Q4: fourth quartile.

Overall, 267 journals were analyzed in the present study, of which the most contributing journal was Oncotarget (136/267, 50.9%), followed by Cancer Medicine (98/267, 36.7%), Journal of Cancer (76/267, 28.5%), Medicine (Baltimore) (72/267, 27.0%), and Cancer Management and Research (69/267, 25.8%). The JCR ranking was retrieved from Web of Science, a global citation database. The top 30 most common journals extracted from the 1566 publications are shown in Figure 5. The distribution of the JCR ranking of the 1566 publications is depicted in Figure 6. A total of 37.4% (585/1566) of the publications were published in the second quartile, followed by the third quartile (489/1566, 31.2%), first quartile (312/1566, 19.9%), unknown JCR ranking (100/1566, 6.4%), and fourth quartile (80/1566, 5.1%).

### International Cooperation

**Figure 7:**
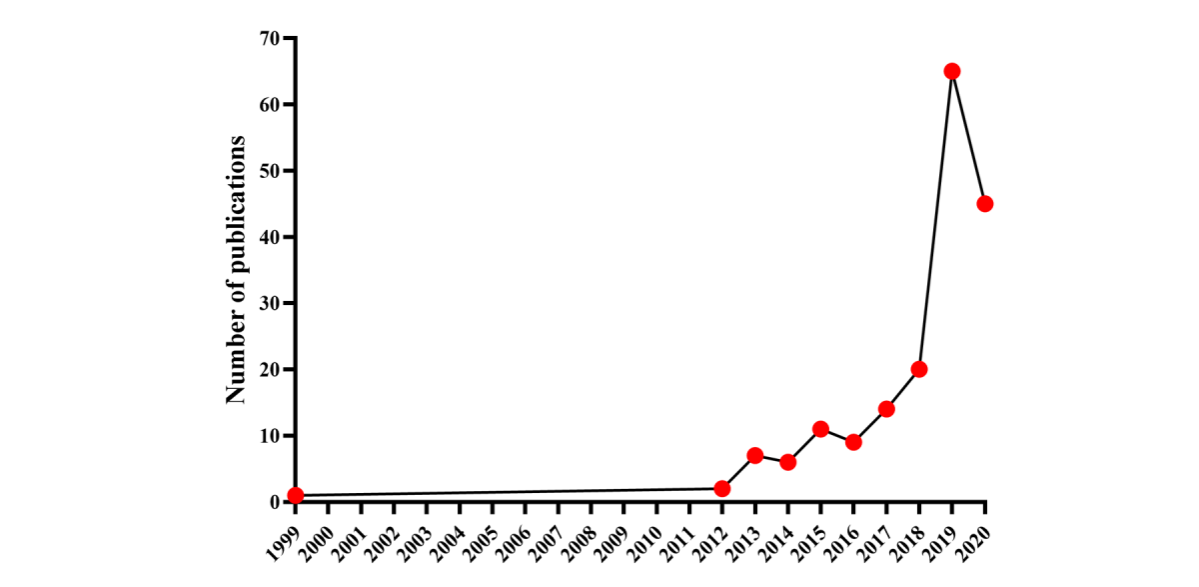
The trends in publications with international cooperation using the Surveillance, Epidemiology, and End Results database between 1999 and 2020.

A total of 180 publications were jointly produced by organizations from China and 23 other countries including the United States (114/180, 63.3%), Russia (17/180, 9.4%), Italy (12/180, 6.7%), Australia (10/180, 5.6%), and Japan (10/180, 5.6%). Chinese institutions first cooperated with institutions from other countries using the SEER database in 1999. Similarly, no papers were jointly produced by institutions from China and other countries utilizing the SEER database during the 11 years from 2000 to the end of 2011. However, the number of papers produced through international cooperation increased substantially from 2016 (9/1566, 0.6%) to 2019 (65/1566, 4.2%). As of August 31, 2020, a total of 45 papers had been published in the year 2020 with international cooperation (Figure 7).

## Discussion

The large sample size of the SEER database and strong statistical guarantees for current cancer concerns add much clinical value to research based on the SEER database. However, the application of China’s big data sector in cancer research is just the beginning. In the recent decade, more and more Chinese scholars have used the SEER database for clinical cancer research. A comprehensive bibliometric study is required to analyze the tendency of Chinese scholars to utilize the SEER database for clinical cancer research and provide a reference for the future of big data analytics. In the present study, we completed a bibliometric study of the SEER database-related publications in China, some prominent characteristics of which were found. First, SEER database-related publications have made continuous and rapid growth in China in recent years. Second, the research topics mainly focused on high-incidence and high-mortality cancers, such as breast cancer, colorectal cancer, lung cancer, gastrointestinal cancer (excluding colorectal cancer), and genital system cancer. Third, publications regarding clinical cancer research using the data from the SEER program were mainly from the best tertiary hospitals in the eastern coastal region of China.

Our study revealed a rapidly growing interest in clinical cancer research from the SEER program in China. With the exception of 1 paper published in 1999, Chinese scholars did not utilize the SEER database for clinical cancer research until 2012. This trend has grown rapidly since 2012, reaching 459 papers in the year 2019. The Covid-19 pandemic has changed almost every aspect of life and society in the year 2020. However, 461 publications were published in the first 8 months of 2020, indicating that the COVID-19 pandemic has not had a negative impact on publications in the SEER database. This phenomenon might attribute to the development of cancer research and informationization in China. Over the past decades, Chinese researchers have gradually made more in-depth use of cancer databases and noticed the important role of the SEER program. This finding was consistent with other studies that have demonstrated an increase in cancer research papers from China [17,18]. In addition, we noticed that not only Chinese scholars, but also scholars from France [19,20], South Korea [21,22], Japan [23], Italy [24], and Switzerland [25], have used the SEER database to conduct clinical cancer research.

With respect to the research topics, the SEER database-related publications were unbalanced, mainly concentrated on breast cancer, colorectal cancer, lung cancer, gastrointestinal cancer, and genital system cancer. Publications on these five cancer sites of the SEER database accounted for 52.3% of all 1566 publications. This regular pattern of distribution of the research topics was consistent with the top 5 cancer sites for estimated cases worldwide for both sexes, which were lung cancer, breast cancer, colorectal cancer, prostate cancer, and stomach cancer [1]. Although these top 5 research topics have been widely studied, Chinese researchers’ interest in them has increased steadily. Moreover, the findings from the SEER studies have been introduced into the treatment guidelines of the National Comprehensive Cancer Network (NCCN) Guidelines and the European Society for Medical Oncology (ESMO) Guidelines, which demonstrate the critical role of the SEER database in clinical cancer research [26,27].

In addition to playing a significant role in the study of these high-incidence cancers, the SEER database is often used to explore rare diseases. Because rare cancers are very uncommon, it is difficult to collect data from a single institution. The SEER database provided substantial valuable data for the study of rare cancers and avoided the selection bias found in single-center retrospective studies. Nevertheless, the results from the SEER database are also retrospective, and prospective studies are needed to validate the results from the SEER database. However, rare cancers are challenging to study prospectively because of their low incidence. The latest NCCN and ESMO guidelines cited several publications on rare cancers based on SEER data, such as male breast cancer, occult breast cancer, and mesenchymal chondrosarcoma [28-30]. Thus, these publications based on SEER data provided insight to help clarify the characteristics, treatment protocols, prognostic indicators, and risk stratification of rare cancers [31,32].

With respect to geographical and institutional distribution, papers published from China using the SEER database were extremely unbalanced. More than 75% of papers were published from the eastern coastal region of China, which was the area with the highest incidence of cancer morbidity in mainland China [33]. In our analysis, more than 90% of publications were produced by the best tertiary hospitals, while 4 of the top 5 contributing organizations were located in the eastern coastal region of China. Regarding the population and economic levels, high-quality medical resources are distributed unevenly in China, with 71 of the 2018 top 100 hospitals in China situated in the eastern coastal region of China [34-37]. In addition, most medical colleges in China are located in this region and could provide resources for cancer research [38]. By relying on universities, researchers at affiliated hospitals can get more research support.

The findings from the SEER program may contribute to cancer prevention and treatment, whether the cancer is common or rare. Nevertheless, the SEER program still has its limitations in that its vital statistics only consist of death and survival data, and there are no data regarding locoregional recurrence and distant metastasis after adopting the corresponding treatment. In addition, the SEER database only represents people who live in the United States. Whether the results of SEER-based research can be applied to other countries, especially to areas of high-incidence cancers like China, needs to be verified by data from other countries. Some Chinese researchers combined the SEER database with their databases to conduct studies and found that the characteristics of the data from the SEER program were consistent with that from Chinese institutions [11-14]. This indicated that the SEER database could provide valuable data for clinical oncology treatment in China. The above results indicate that clinical cancer research based on the SEER database may provide a valuable reference for clinical cancer researchers in China.

China should establish cancer databases based on its demographic characteristics. To our knowledge, Chinese cancer registration work was started much later than in other upper middle-income countries [4]. The number of cancer registries in China has increased rapidly since 2002, while a significant gap still exists between China and other upper middle-income countries. At present, the items and contents of population-based cancer data in China are limited. Therefore, high-quality cancer databases based on Chinese demographic data are urgently needed to better reflect the oncology practices in China [39-42].

The limitation of our analysis is that we only collected publications from the PubMed database, which may ignore some publications that were not indexed in PubMed, resulting in incomplete data.

### Conclusions

In conclusion, our study suggests that clinical cancer research regarding the SEER database has rapidly increased in China in the past decade. High-quality and national comprehensive cancer registries from China are needed to provide a reference for the future of big data analytics.

## Abbreviations

ESMO: European Society for Medical Oncology
JCR: Journal Citation Reports
NCCN: National Comprehensive Cancer Network
SEER: Surveillance, Epidemiology, and End Results
